# Impact of early initiation of PCSK9I monoclonal antibodies after acute coronary syndrome

**DOI:** 10.1016/j.ihj.2026.04.013

**Published:** 2026-04-20

**Authors:** Pravin K. Goel, Aditya Kapoor, Kala Jeetender Jain, Rajesh Thachathodiyl, Vikas Reddy, Vivek Guleria, Neeraj Bhalla, Shyam Sasidharan

**Affiliations:** a Department of Cardiology, Medanta Super Speciality Hospital, A Pocket, 1, Amar Shaheed Path, Golf City, Muzaffar Nagar Ghusval, Lucknow, Uttar Pradesh, 226002, India; b Department of Cardiology, Sanjay Gandhi Postgraduate Institute of Medical Sciences New PMSSY Rd, Sanjay Gandhi PGIMS, Raibareli Rd, Lucknow, Uttar Pradesh, 226014, India; c Department of Cardiology, Yashoda Hospitals, Survey No. 41/14, JNTU, To, Hitech City Main Rd, Khanamet Village, Sharilimgampally, Kothaguda, Hyderabad, Telangana, 500081, India; d Department of Cardiology, Amrita Hospital, Amrita Hospital Ponekkara Rd, P. O, Edappally, Ernakulam, Kochi, Kerala, 682041, India; e Department of Cardiology, Health Valley Hospital, Health Valley Hospital Door No. 1-111/4/46 & 48/E, Beside NISUM Khanamet Road, Kondapur, HITEC City, Hyderabad, Telangana, 500084, India; f Department of Cardiology, Command Hospital, Command Hospital Chandimandir Cantonment, Panchkula, Haryana, 134107, India; g Department of Cardiology, BLK-Max Hospital, BLK-Max Super Speciality Hospital Pusa Rd, Radha Soami Satsang, Rajinder Nagar, New Delhi, Delhi, 110005, India; h Department of Cardiology, KIIMS Hospital, KIMSHEALTH Hospital 1, Vinod Nagar Rd, Anayara, Thiruvananthapuram, Kerala, 695029, India

**Keywords:** LDL-C, Acute coronary syndrome, Atherosclerotic cardiovascular disease, Early initiation, PCSK9 inhibitors

## Abstract

Low-density lipoprotein cholesterol (LDL-C) is a key modifiable risk factor for atherosclerotic cardiovascular (CV) disease in patients with acute coronary syndrome (ACS). While high-intensity statins are foundational, many patients fail to reach guideline-recommended targets, leaving a high residual risk during the early post-ACS phase. Evidence from EVOPACS and EVACS trials demonstrates that early in-hospital proprotein convertase subtilisin/kexin type 9 inhibitor (PCSK9i) initiation achieves rapid, intensive LDL-C lowering, with over 90% of patients attaining targets of ≤1.4 mmol/L by discharge. Furthermore, the PACMAN-AMI and HUYGENS trials confirm that PCSK9i therapy promotes plaque regression and stabilisation by increasing fibrous cap thickness and reducing lipid-rich necrotic core content. Meta-analyses indicate significant reductions in major adverse CV events and ACS-related hospitalisations within 6–18 months. Early PCSK9i integration offers a potent, safe strategy to bridge lipid management gaps and optimize secondary prevention outcomes in high-risk populations, including those in India.

## 1 Introduction

Acute coronary syndrome (ACS) usually results from the rupture or erosion of an unstable atherosclerotic plaque, leading to partial or complete coronary thrombosis and ischaemia. ACS comprises three closely related conditions of varying severity—unstable angina, non-ST–elevation myocardial infarction (NSTEMI), and ST-segment elevation myocardial infarction (STEMI).^1^ Patients with ACS are at the greatest risk for cardiovascular (CV) complications during the acute phase. Although appropriate management reduces this risk over time, an elevated risk of recurrent CV events persists for months to years following the index event.^1^

ACS remains a major public health concern in India, with a growing burden among middle-aged adults. Recent data from the RECent trends in the pattern and lOng-term management stRategy of patients Diagnosed with Acute Coronary Syndrome (RECORD ACS-2) study highlighted that the highest proportion of ACS cases (39%) occurred in individuals aged 51–60 years.^2^ Additionally, the long-tErm follow-up of antithrombotic management Patterns In acute CORonary syndrome patients (EPICOR)-India study, part of the EPICOR registry, examined 2468 Indian ACS patients and revealed key challenges, including delays in early care, suboptimal adherence to guideline-recommended therapies, and a 2-year mortality rate of 6.7%.^3^ These findings underscore the need for enhanced acute and long-term management strategies, particularly the optimisation of lipid-lowering therapies, in this high-risk population.

Early low-density lipoprotein cholesterol (LDL-C) reduction in high-risk ACS patients offers CV protection by stabilising plaques and lowering the risk of recurrent events during the vulnerable post-ACS period. Individuals with a history of myocardial infarction (MI) remain at high risk for adverse CV outcomes, including recurrent MI, heart failure (HF), stroke, and CV mortality.^4^ The median time to recurrent MI extends to approximately 4.5 months in patients with left ventricular dysfunction or HF, with the highest risk of recurrent events and mortality concentrated in the initial three months post–MI.^5^ This residual risk persists despite the implementation of contemporary secondary prevention strategies and is influenced by several clinical variables, including advanced age, diabetes mellitus, prior cardiovascular disease (CVD), and incomplete revascularisation.

Prognosis in ACS is further influenced by the extent of myocardial damage and the burden of comorbidities.^4^ In extensive post–MI patient populations characterised by a high prevalence of comorbidities, including hypertension, dyslipidaemia, type 2 diabetes mellitus, and chronic kidney disease, nearly one-quarter develop new-onset HF, most frequently occurring within the initial three months following the infarction.^4^ In the VALIANT (Valsartan in Acute Myocardial Infarction Trial) cohort, 9.6% of patients experienced a recurrent MI within a median of 136 days. One-year mortality was 10.3% overall, but increased to 38.3% among those with a recurrent MI.^5^

### 1.1 The window of vulnerability: the first three months post–MI

Approximately one-third of recurrent acute MIs occur within the first 30 days of the index event, a period associated with particularly elevated mortality risk.^5^ The initial three months post–MI represent a period of heightened vulnerability, during which the risk of recurrent CV events is markedly elevated. In this context, early initiation of intensive lipid-lowering therapy (LLT) is warranted to facilitate the prompt attainment of LDL-C targets and mitigate the risk of subsequent events in patients with ACS. The principle of the “strike early and strike strong” strategy is supported by growing evidence demonstrating that prompt administration of potent LLT, such as PCSK9 inhibitors, during hospitalisation or the immediate post-discharge period leads not only to rapid and substantial LDL-C reductions but also to favourable modifications in coronary plaque morphology.^6^

In a Korean cohort of over 16,000 atherosclerotic cardiovascular disease (ASCVD) patients, achieving LDL-C <55 mg/dL within 4–12 weeks post-discharge was linked to a significantly lower risk of recurrent major CV events. The benefit was particularly pronounced in individuals with recent ACS. Furthermore, early LDL-C goal attainment resulted in a 19% reduction in CV hospitalisations and a 31% reduction in the average length of stay.^7^ These findings reinforce the rationale for initiating intensive LLT early in the post–MI period, with particular attention to LDL-C goal attainment in very high-risk patients.

### 1.2 LDL-C as a key driver of recurrent CV events

In the early phase following ACS, significant alterations in lipid metabolism occur. LDL-C and high-density lipoprotein (HDL) levels decline while triglyceride concentrations increase, a pattern associated with heightened CV risk. ACS is also characterised by a pro-inflammatory milieu, with elevated levels of inflammatory biomarkers such as C-reactive protein (CRP), which serves as an independent predictor of recurrent CV events.^8^ In a cohort of 47,884 post-percutaneous coronary intervention (PCI) patients, only 57% achieved LDL-C <70 mg/dL within six months despite high-intensity statin (HIS) therapy. Over a median follow-up of 3.2 years, CV event rates increased with higher LDL-C levels: 55.2 (<70 mg/dL), 60.3 (70–100 mg/dL), and 94.0 (≥100 mg/dL) per 1000 person-years. This reinforces the importance of intensive lipid lowering.^9^

Although plasma LDL-C levels may initially decline following an ACS event, this decline is transient and may be misleading. During the acute phase, hepatic LDL synthesis increases, and LDL particles become smaller and denser. These changes enhance their atherogenicity and susceptibility to oxidative modification. Such compositional alterations begin early after infarction, with LDL particle size continuing to evolve over the following months, further contributing to residual CV risk.^8^

Following an MI, atherosclerotic plaque progression may accelerate, driven by increased protease activity, oxidative stress, and sustained inflammation. These changes contribute to plaque instability, particularly in lesions with a thin fibrous cap, a large lipid core, and dense inflammatory infiltrates, features that predispose the plaque to rupture. Rupture of plaques is implicated in most ACS episodes.^10^

As noted previously, despite statin therapy, LDL-C targets are not consistently achieved in clinical practice. In the Swedish Web-System for Enhancement and Development of Evidence-Based Care in Heart Disease Evaluated According to Recommended Therapies (SWEDEHEART) registry (n = 56,262 patients with MI), those in the lowest quartile of non-HDL–C at one year had a 24% lower risk of major adverse CV events (Hazard ratio [HR] 0.76, 95% confidence interval [CI] 0.71–0.81). Achieving the lowest non-HDL–C levels at both two months and one year after MI was linked to improved outcomes, with the greatest risk reduction seen in patients who reached target levels within two months and maintained them over time. These findings reinforce the need for intensified lipid-lowering strategies in the high-risk post-ACS setting. In a real-world, multicentre Indian study (n = 575) on HIS, only 20.9% of patients reached LDL-C <55 mg/dL, and 55.7% remained above 70 mg/dL one year after ACS.^11^ Another cross-sectional, single-centre Indian study in 403 ACS patients demonstrated an atherogenic lipid profile, with 43.2% having elevated LDL-C levels (>130 mg/dL).^12^ Given the high early recurrence rates, limitations of current LDL-C lowering with statins alone, and delayed achievement of guideline-recommended lipid targets, there is growing interest in the early initiation of non-statin LLTs. LDL-C is a key modifiable risk factor in ASCVD, and early aggressive lipid lowering is crucial for high-risk patients, especially those with prior CV events.

Proprotein convertase subtilisin/kexin type 9 inhibitor (PCSK9i), which offers rapid and potent LDL-C reduction, has emerged as a compelling option in this setting. This review explores the evidence, rationale, and clinical considerations for early initiation of PCSK9 monoclonal antibodies (mAbs) following ACS, particularly during the vulnerable early post–MI period.

### 1.3 Challenges in achieving LDL-C targets with traditional statin-based therapies

Statins lower the risk of major vascular events and vascular mortality, with an estimated 20% risk reduction for each 1 mmol/L decrease in LDL-C. Further reductions in LDL-C yield progressively greater CV benefits.^13^ Accordingly, early and effective LDL-C management in the acute setting is vital to limit additional vascular injury and prevent recurrent events.

Despite their established efficacy, statin monotherapy may be insufficient to achieve guideline-recommended LDL-C targets within the narrow high-risk window following ACS. Even with structured follow-up every 4–6 weeks, it may take up to three months to attain LDL-C goals using statins alone, overlapping with the period of greatest vulnerability for recurrent CV events. Therefore, an early and intensive lipid-lowering approach, including immediate statin initiation and dual therapy, is recommended for all ACS patients.^14^

## 2 Lipid-lowering agents beyond statins

While statins remain the foundation of LLT in the management of ACS, a significant proportion of patients fail to reach target LDL-C levels with statins alone. Additional therapies with ezetimibe and bempedoic acid may be used in patients with a suboptimal response or statin intolerance. Current guidance supports the immediate initiation of statins, ideally during hospitalisation, and recommends the routine consideration of combination therapy (e.g., with ezetimibe or a PCSK9i) in patients with ACS to expedite LDL-C goal attainment.^6^ The concept of combination therapy has gained traction, particularly in high-risk patients such as those with ACS, where early and aggressive LDL-C lowering is critical.

### 2.1 Elevated PCSK9 levels and ACS

In patients with ACS, serum PCSK9 levels are elevated, driven by cardiac ischaemia, contributing to plaque instability, inflammation, and thrombosis.^15^ PCSK9 exerts direct proinflammatory effects and interacts with several molecular targets involved in immune and vascular regulation, such as lectin-like oxidised LDL receptor-1 (LOX1), apolipoprotein E receptor 2 (apoER2), cluster of differentiation 36 (CD36), and low-density lipoprotein receptor-related protein 1 (LRP1). By promoting the degradation of apoER2, PCSK9 attenuates the anti-inflammatory effects of apoE/apoER2 signalling, thereby facilitating a proatherogenic milieu.^16^ Elevated PCSK9 levels also stimulate dendritic cell maturation and drive naïve CD4^+^ T cells toward proinflammatory Th1 and Th17 types, leading to increased production of IFN-γ and IL-17 A.^17^ Additionally, PCSK9 has been implicated in platelet activation, further enhancing thrombotic risk.^15^ Collectively, these mechanistic insights support the therapeutic potential of PCSK9i in mitigating residual inflammatory and thrombotic risk in ACS.

### 2.2 The role of PCSK9i in the early intensification of lipid treatment

PCSK9i play a pivotal role in the early intensification of lipid-lowering strategies following ACS. Early initiation of PCSK9i within 48 h of ACS presentation has been associated with significant reductions in the risk of MI (Relative risk [RR] 0.59), ACS-related hospitalisation (RR 0.53), and need for revascularisation over a six-to 18-month follow-up period (RR 0.59). Early therapy also shows favourable trends in stroke, CV events, and all-cause mortality, accompanied by marked reductions in total cholesterol, LDL-C, apolipoprotein B, and lipoprotein(a) within the first 4–12 weeks.^18^ When added early in the post-ACS period to a regimen including HIS and ezetimibe, PCSK9i allow for more rapid and pronounced LDL-C lowering, which may be critical in reducing early recurrence risk.^18^

Two PCSK9 mAbs, evolocumab^19^ and alirocumab,^20^ are approved by the United States Food and Drug Administration as adjunctive agents for managing high-risk patients, including those with recent ACS. In patients with acute MI, administration of a single 140 mg dose of evolocumab at the time of PCI resulted in significantly greater reductions in LDL-C and a higher rate of early target LDL-C achievement compared to those who did not receive evolocumab.^21^ Furthermore, PCSK9i therapy has been associated with reduced early hospital readmissions following ACS, suggesting a broader benefit in post-discharge outcomes.^18^

#### 2.2.1 Pleiotropic effects of PCSK9i mAbs: Beyond LDL-C lowering

Beyond LDL-C lowering, PCSK9i, particularly evolocumab, may confer additional CV benefits through pleiotropic effects. These include anti-inflammatory actions, reductions in cytokine and plaque destabilisation markers, enhanced endothelial function, and promotion of plaque stabilisation. Evolocumab also modestly lowers Lp(a), may increase HDL levels, and exhibits anti-aggregatory and anticoagulant effects, further supporting its utility in comprehensive atherosclerotic disease management.^22^

#### 2.2.2 Unique pharmacokinetics of PCSK9i mAbs

Evolocumab is a human mAb that targets PCSK9 on liver cells, resulting in significant LDL-C reduction. It has a half-life of 11–17 days, with maximal PCSK9 suppression occurring within 4 h and peak LDL-C reduction (55%–75%) achieved within 1–2 weeks.^19^ Alirocumab, another PCSK9 mAb, has a median half-life of 17 to 20 days at a steady state.^20^ The pharmacokinetic profiles of both evolocumab and alirocumab are consistent across diverse patient populations, with factors such as age, gender, race, body weight, and renal function having no significant impact on drug exposure. Consequently, no dose adjustments are required based on these parameters.^23^

### 2.3 Peri-PCI benefits of PCSK9 inhibition in ACS

Early initiation of PCSK9i, particularly evolocumab, in the peri-PCI setting offers potential benefits in patients with ACS by rapidly lowering LDL-C and exerting anti-inflammatory and plaque-stabilising effects. In a pilot trial of 124 post-PCI ACS patients, three-month PCSK9 antibody therapy significantly reduced LDL-C levels (from ∼139 to 37 mg/dL), with comparable one-year CV event rates in the PCSK9 group (8.1%) and the conventional therapy group with statins (6.7%).^24^

The Kerala ACS study, involving 25,748 patients, reported in-hospital mortality and major adverse cardiovascular events (MACE) rates of 8.2% and 10.3% in patients with STEMI, along with suboptimal discharge medication use (<80%), highlighting the importance of early and intensive LDL-C lowering in Indian patients with ACS.^25^ The ODYSSEY OUTCOMES trial, involving nearly 19,000 post-ACS patients, demonstrated a 15% relative reduction in major CV events and all-cause mortality over a median of 2.8 years with alirocumab added to HIS.^26^

### 2.4 Comparative efficacy of PCSK9i: evidence from clinical trials

#### 2.4.1 Evolocumab in combination with LLT

In a pilot trial of ACS patients undergoing PCI, 124 individuals were randomised to receive either short-term PCSK9 antibody therapy with conventional LLT or LLT alone. The primary composite outcome, death, MI, stroke, unstable angina, or revascularisation, occurred in 9.7% and 14.5% of patients receiving vs not receiving PCSK9 antibodies, respectively (HR 0.70; 95% CI 0.25–1.97; p = 0.498).^24^

The pivotal FOURIER trial demonstrated that evolocumab plus statins led to a substantial 59% reduction in LDL-C levels and a 15% relative risk reduction in major CV events over a median follow-up of 2.2 years.^27^ Subgroup analyses showed enhanced benefits in patients with recent or multiple MIs or multi-vessel coronary disease, with relative risk reductions up to 30%.^28^

Long-term safety and efficacy were assessed in the FOURIER OLE, OSLER-1, and OSLER-2 open-label extension studies involving patients with hypercholesterolaemia. For up to five years, evolocumab added to standard therapy achieved sustained ∼60% LDL-C reductions and was well tolerated, with no new safety concerns. Fewer events were observed in the evolocumab group, supporting its long-term use in high-risk patients. Crucially, FOURIER-OLE highlights a ‘legacy effect’. Patients achieving target LDL-C levels early in the post-ACS course demonstrated a sustained divergence in event curves compared to those with delayed intensive treatment.^29^

#### 2.4.2 Evolocumab vs. other non-statin therapies

Evolocumab demonstrates rapid LDL-C lowering within 4–7 days (65%–70%), surpassing the more modest reductions seen with ezetimibe (∼12%), bempedoic acid (∼18%), and inclisiran (∼45% over 12 weeks).^30^, ^31^, ^32^, ^33^, ^34^ This rapid and potent effect makes evolocumab particularly useful in high-risk clinical settings where early LDL-C reduction is critical. The DESCARTES trial further supports its consistent efficacy in LDL-C lowering across patients with different CV risk profiles and background therapies.^35^

#### 2.4.3 Evolocumab vs. placebo

In the GLAGOV study of statin-treated patients with coronary atherosclerosis, monthly evolocumab significantly reduced LDL-C levels and induced regression of coronary plaque burden compared to placebo over 76 weeks.^36^ Notably, the percentage of atheroma volume decreased by 0.95% with evolocumab but slightly increased in the placebo group, indicating plaque stabilisation and regression. These imaging findings provide mechanistic insights into the CV benefits of evolocumab observed in clinical outcome trials.

#### 2.4.4 Evolocumab in combination with statins

Real-world data corroborate the clinical benefits of adding evolocumab to statins. One cohort showed a significant reduction in ischaemic stroke, CV death, MI, and related hospitalisations compared to controls.^31^ Early use of evolocumab during the ACS hospitalisation (EVOPACS) trial led to rapid and substantial LDL-C lowering with good tolerability, supporting its role in acute settings.^37^ Similarly, the EVACS trial showed that a single dose of evolocumab in NSTE-ACS patients reduced LDL-C at one month versus placebo.^38^ Moreover, the HUYGENS trial demonstrated improvements in coronary plaque morphology and stabilisation when evolocumab was combined with HIS after NSTEMI, including increased fibrous cap thickness and reduced lipid content, key markers of plaque vulnerability.^39^

The EARLY-STEMI trial (TCT-68) extended these findings using evolocumab in STEMI, demonstrating significant LDL-C reduction and reduced carotid intima-media thickness with no treatment-limiting adverse events.^40^

A network meta-analysis by Toth et al comparing lipid-lowering therapies across 69 randomised trials found that PCSK9i added to medium-to high-intensity statin therapy reduced LDL-C by 54%–74% versus placebo and 26%–46% versus ezetimibe, with a significant treatment difference favouring evolocumab over alirocumab. Adverse event rates were similar across all comparators.^41^ These results were further supported by Justino et al. (6 RCTs, n = 996), confirming significant reductions in LDL-C, Lp (a), and other atherogenic lipoproteins with early PCSK9i versus placebo. No increase in adverse events was noted.^42^ Hosseini et al. (9 RCTs and 3 cohort studies) evaluated early PCSK9i initiation within 48 h of hospitalisation in patients with ACS, reporting significant reductions in MI, ACS-related hospitalisation, and revascularisation at 6–18 months, along with improved lipid control at 4–12 weeks.^18^

#### 2.4.5 Alirocumab vs. placebo and in combination with statins

Alirocumab, another PCSK9i, showed a 64 mg/dL LDL-C reduction by day 14 post-NSTEMI in the VCU-AlirocRT trial without significant safety concerns.^18^ The PACMAN-AMI and EPIC STEMI trials further showed significant coronary plaque regression and LDL-C lowering with alirocumab plus HIS in acute MI and STEMI settings, respectively.^18^^,^^43^ Notably, EPIC STEMI highlighted that LDL-C benefits were lost upon drug discontinuation.^43^ Table 1 presents key clinical trials of PCSK9i, highlighting their effects on LDL-C reduction, CV outcomes, and safety across different patient groups and treatment settings.

**Table 1:** Key clinical trials evaluating the efficacy, safety, and tolerability of PCSK9i in ACS.

Trial	Population	Intervention	Duration	Primary Outcomes	Key Efficacy Results	Safety & Tolerability
**FOURIER (2017)**^27^^,^^28^^,^^31^	Patients with ASCVD	Evolocumab + statin vs. placebo + statin	Median 2.2 years	Composite of CV death, MI, stroke, unstable angina, hospitalisation, or coronary revascularisation	59% LDL-C reduction; 15% relative risk reduction in major CV events	Well tolerated; similar adverse event rates between both groups, including new-onset diabetes and neurocognitive effects
**FOURIER OLE** **OSLER 1 & 2 (long-term extension)**^29^	Patients with hypercholesterolaemia	Evolocumab + standard therapy vs. standard therapy alone	Up to 5 years	Long-term safety and efficacy	Sustained ∼60% LDL-C reduction; no new safety signals	Low serious adverse events; well tolerated
**DESCARTES (2014)**^35^	Patients with varying CV risk	Evolocumab + diet/statin vs. placebo + diet/statin	52 weeks	LDL-C reduction	Reduction in LDL-C by 48.5% to 61.6% across backgrounds of diet alone, atorvastatin (10 mg or 80 mg), and atorvastatin plus ezetimibe (p < 0.001)	No significant safety concerns; balanced adverse events
**GLAGOV (2016)**^36^	Statin-treated patients with coronary atherosclerosis	Evolocumab + statin vs. placebo + statin	76 weeks	Change in coronary atheroma volume	Significant plaque regression vs. placebo (64.3% vs. 47.3%); PAV reduced by 0.95%	Safety is consistent with other PCSK9i studies
**EVOPACS (2019)**^37^	ACS patients not at LDL-C goal, including STEMI and NSTE-ACS (n = 308)	Evolocumab (420 mg) within ≤24 h of randomisation + HIS vs. placebo + HIS	8 weeks	Percent change in LDL-C from baseline to week 8	LDL-C reduced from 3.61 to 0.79 mmol/L (evolocumab) vs. 3.42 to 2.06 mmol/L (placebo); additional reductions in ApoB, total cholesterol, non-HDL-C	No significant increase in adverse events; well tolerated in the acute ACS setting
**EVACS (2020)**^38^	NSTEMI patients with troponin-I ≥5 ng/mL on high-intensity statin therapy (n = 57)	Evolocumab 420 mg (single dose) within 24 h of presentation vs. placebo	30 days	Change in LDL-C from baseline to 30 days	LDL-C 28.6 mg/dL lower in evolocumab vs. placebo at 30 days (p < 0.0001); 80.8% vs. 38.1% achieved AHA/ACC LDL target at hospital discharge	No significant difference in adverse or serious adverse events between groups
**HUYGENS (2021)**^39^	NSTE-ACS patients post-PCI (n = 150)	Evolocumab (420 mg monthly) vs. placebo for 48 weeks post-PCI	50 weeks	Minimum fibrous cap thickness by OCT at 50 weeks	Greater increase in min. Fibrous cap thickness (+42.7 vs. +21.5 μm); reduction in max. Lipid arc (−57.5° vs. −31.4°) and macrophage index (−3.17 vs. −1.45 mm)	Well tolerated; no significant safety concerns
**EARLY-STEMI (2025)**^40^	STEMI patients post-primary PCI (n = 142, interim analysis)	Evolocumab (140 mg) within 48 h of successful PCI + standard therapy vs. standard therapy alone	12 months	LDL-C reduction and safety outcomes at 12 months	Significantly greater LDL-C reduction at all time points (p < 0.05); reduced carotid intima-media thickness (p < 0.04); MACE 8.6% vs. 16.7% (non-significant)	No treatment-limiting adverse events; well tolerated
**ODYSSEY OUTCOMES (2018)**^26^	Post-ACS patients with elevated LDL-C despite statins	Alirocumab (75 mg q2w) + HIS vs. placebo + HIS	Median 2.8 years	Major CV events (CV death, MI, stroke, unstable angina, revascularisation)	15% relative risk reduction in major CV events	Well tolerated; no significant differences in adverse events; injection site reactions comparable (3.8% vs. 2.1%)
**PACMAN-AMI (2022)**^44^	Patients post-acute MI	Alirocumab + HIS vs. placebo + HIS	52 weeks	Coronary plaque regression in non-infarct arteries	Significantly greater plaque regression with alirocumab; mean change in % PAV (−2.13% vs −0.92%)	Injection site reactions (6.1% vs. 3.3%), neurocognitive events (2.0% vs. 0%), allergic reactions (3.4% vs. 0%)
**VCU-AlirocRT (2019)**^45^	NSTEMI patients	Alirocumab vs. placebo	14 days	LDL-C reduction	64 mg/dL LDL-C reduction with alirocumab vs. a minimal change in placebo group	No significant CRP changes or serious adverse events
**EPIC STEMI (2021)**^43^	STEMI patients undergoing PCI	Alirocumab + HIS vs. placebo + HIS	6 weeks	LDL-C reduction	22% greater LDL-C reduction with alirocumab (73% vs. 48%)	No unexpected safety issues

ACS: Acute coronary syndrome; CRP: C-reactive protein; CV: Cardiovascular; HIS: High-intensity statin; LDL-C: Low-density lipoprotein cholesterol; MI: Myocardial infarction; NSTEMI: Non-ST–segment elevation myocardial infarction; PCI: Percutaneous coronary intervention; PAV: Percent atheroma volume; PCSK9i: Proprotein convertase subtilisin/kexin type 9 inhibitor; q2w: Every two weeks; STEMI: ST-segment elevation myocardial infarction.

#### 2.4.6 Small interfering RNA (siRNA)-based PCSK9 inhibition: mechanistic distinctions and emerging evidence in ACS

Among emerging therapies, the siRNA-based PCSK9i represents a novel and mechanistically distinct class within the broader PCSK9i landscape. Inclisiran, an siRNA, exerts its lipid-lowering effect by reducing hepatic PCSK9 mRNA synthesis. The ORION-10 and ORION-11 phase III trials demonstrated an approximately 50% reduction in LDL-C with a twice-yearly dosing schedule in patients with elevated LDL-C on background statin therapy.^32^ This dosing may improve adherence as compared to the fortnightly injections required with monoclonal antibodies. A pilot study confirmed early LDL-C reductions with inclisiran initiated within one month of ACS hospitalisation.^46^ However, evidence in the acute ACS setting remains limited. Until CV outcomes data mature, PCSK9i monoclonal antibodies remain the preferred evidence-based options for lipid-lowering intervention in ACS.

Together, these trial data provide the foundation for a robust early initiation strategy, discussed in the following section.

### 2.5 Early PCSK9i initiation: Maximising therapeutic benefit in ACS

Evidence from trials like EVOPACS,^37^ EVACS,^38^ and EARLY-STEMI^40^ demonstrates that early in-hospital administration is not only safe but strongly warranted for achieving the stringent ESC/EAS LDL-C targets (≤1.4 mmol/L) that are frequently missed with statin monotherapy alone. Beyond rapid LDL-C lowering, the pleiotropic effects observed in the HUYGENS and PACMAN-AMI^44^ trials, specifically the increase in fibrous cap thickness and reduction in lipid-rich necrotic cores, suggest that early intervention may physically stabilise vulnerable plaques during the highest-risk period following an index event. This proactive strategy addresses the residual inflammatory and cholesterol risk immediately, potentially preventing the early recurrent ischemic events that characterise the post-ACS phase. Table 2 summarises the mechanistic insights on the benefits of early PCSK9 inhibition in ACS.

**Table 2:** Clinical evidence supporting early PCSK9 inhibition in ACS^37^, ^38^, ^39^, ^40^^,^^44^.

Trial	Agent	Timing of initiation	Key mechanistic benefit
EVOPACS	Evolocumab	<24 h of ACS	Rapid LDL-C reduction to target values during the acute hospital phase
EARLY-STEMI	Evolocumab	<48 h of STEMI	Demonstrated significant LDL-C reduction, decreased CIMT, and a favourable safety profile
EVACS	Evolocumab	<24 h of NSTEMI	Rapid achievement of LDL-C targets by discharge with a single dose
HUYGENS	Evolocumab	In-hospital (NSTEMI)	Stabilised vulnerable plaque by increasing the minimum fibrous cap thickness
PACMAN-AMI	Alirocumab	<24 h of AMI	Significant plaque regression and increased fibrous cap thickness
EPIC STEMI	Alirocumab	Pre-primary PCI	Achievement of >90% target within the ultra-acute window

ACS: Acute coronary syndrome; AMI: Acute myocardial infarction; CIMT: Carotid intima-media thickness; LDL-C: Low-density lipoprotein cholesterol; NSTEMI: Non-ST-elevation myocardial infarction; PCI: Percutaneous coronary intervention; STEMI: ST-segment elevation myocardial infarction.

#### 2.5.1 High-risk groups warranting early intensification of lipid treatment

Despite existing recommendations, many high-risk individuals remain undertreated. In the large real-world VESALIUS-REAL cohort study involving 195,621 high-risk individuals with established atherosclerosis and/or diabetes mellitus, 69% were not receiving any LLT despite having LDL-C levels above recommended targets.^47^ This treatment gap emphasised the need for early and intensive lipid management in these populations.

Against this backdrop, current evidence supports a stepwise but proactive approach to lipid intensification. For patients with baseline LDL-C >100 mg/dL, combination therapy with statins and ezetimibe is recommended. If LDL-C remains above 70 mg/dL despite maximally tolerated statins and ezetimibe, PCSK9i should be considered within three months, especially in high-risk individuals with diabetes mellitus. Initiation is strongly advised when LDL-C persists at or above 100 mg/dL. Early use of PCSK9i is also recommended in patients with recent ACS, recurrent ischaemic events, multi-vessel or poly-vascular disease, or symptomatic peripheral artery disease.^48^

In individuals with multiple CV risk factors, PCSK9i offer an effective strategy for intensifying lipid management. A network meta-analysis of 84 randomised controlled trials involving over 246,000 patients demonstrated that while statins rank highest in reducing CV events and mortality, PCSK9i achieve the greatest LDL-C reductions. Both therapies provide comparable CV benefits. Importantly, PCSK9i do not increase the risk of statin-associated side effects such as elevated alanine aminotransferase, creatine kinase levels, or new-onset diabetes mellitus.^49^ Among patients with ACS and multi-vessel disease undergoing PCI, early initiation of evolocumab, along with statin therapy, resulted in significant reductions in LDL-C levels and the risk of recurrent CV events.^50^

#### 2.5.2 Challenges in adherence to PCSK9 therapy

Despite the proven efficacy of PCSK9 inhibitors, real-world adherence remains a significant challenge. Adherence to PCSK9i over time was suboptimal despite reimbursement support, reflecting the need for patient engagement and system-level solutions to improve long-term use. In a nationwide Norwegian study of 4784 PCSK9 mAb users, 17% had significant treatment gaps within three years, and over half discontinued statins, while 40% stopped ezetimibe after starting PCSK9 therapy. This highlights a substantial opportunity to improve adherence to oral LLT in high-risk patients initiating PCSK9i.^51^

### 2.6 Current guidelines and recommendations

The 2025 American Association of Clinical Endocrinology (AACE) recommends statins as the first-line treatment for dyslipidaemia and conditionally advises a treatment goal of LDL-C <70 mg/dL in individuals with or at increased risk of ASCVD. PCSK9 mAbs are conditionally recommended in these populations, with treatment decisions guided by clinical judgement and patient-specific factors.^52^ In adults with dyslipidaemia on maximally tolerated statin therapy who have ASCVD or are at elevated risk and have not reached the LDL-C goal of <70 mg/dL, the AACE conditionally recommends adding evolocumab or alirocumab to standard treatment.^53^

As per the 2023 European Society of Cardiology (ESC)guidelines, if LDL-C targets are not reached within 4–6 weeks despite maximally tolerated statin therapy and ezetimibe, the addition of a PCSK9i is recommended.^14^ Similarly, the 2025 ACC/AHA/ACEP/NAEMSP/SCAI guidelines recommend considering the addition of non-statins such as ezetimibe, PCSK9i, inclisiran, or bempedoic acid when LDL-C levels remain ≥70 mg/dL despite maximal statin therapy.^1^ Further intensification can be considered if LDL-C levels range between 55 mg/dL and <70 mg/dL in high-risk populations.^1^ These recommendations collectively reinforce the role of evolocumab as an effective intensification strategy for high-risk patients who do not achieve LDL-C targets despite maximal statin and ezetimibe therapy, particularly following ACS or MI.

Clinicians may consider a “bolus and chase” strategy, involving an initial dose of a PCSK9i followed by periodic LDL-C monitoring to guide further dosing. However, this approach, used selectively in post-ACS settings, is not guideline-recommended and requires further validation. In India, catastrophic health spending affects 50% of patients following CVD hospitalisation.^54^ While pharmacotherapy entails ongoing costs, it remains more cost-effective and clinically preferable than recurrent hospitalisations due to preventable CV events. Table 3 summarises the key considerations for initiating, dosing, and monitoring PCSK9i in patients with ACS, highlighting when and how to use them for optimal lipid-lowering outcomes.

**Table 3:** How and when to use PCSK9i in ACS management.

Clinical consideration	Recommendations
**Initiation**	• Begin in high-risk patients, especially post-ACS, if LDL-C remains above target despite maximally tolerated statins ± ezetimibe. • Early initiation after ACS helps achieve rapid LDL-C reduction and stabilise plaques.
**Dosing and frequency**	• ***Evolocumab*:** 140 mg every 2 weeks or 420 mg monthly. • ***Alirocumab*:** 75–150 mg every 2 weeks or 300 mg monthly. • Dose adjustments should be guided by LDL-C levels and clinical response.
**Use during peri-PCI**	• Initiate PCSK9i during the peri-PCI period to accelerate LDL-C lowering and improve plaque stability. • Use alongside standard LLT for optimised care.
**Monitoring and follow-up**	• Regular LDL-C measurement every 4–12 weeks post-initiation. • Adjust therapy based on LDL-C targets, patient adherence, and treatment tolerance.

ACS: Acute coronary syndrome; LDL-C: Low-density lipoprotein cholesterol; LLT: Lipid-lowering therapy; PCI: Percutaneous coronary intervention; PCSK9i: Proprotein convertase subtilisin/kexin type 9 inhibitor.

## 3 Conclusion

Early lipid-lowering intervention in post–MI patients is vital to reducing the risk of recurrent CV events. Personalised treatment strategies that account for individual risk profiles are essential, particularly in high-risk populations, including those with multi-vessel disease, diabetes mellitus, or persistently elevated LDL-C levels despite optimal statin therapy. While statins remain the foundation of LLT, established non-statin options like PCSK9 mAbs have expanded the therapeutic landscape, offering effective strategies for optimising post–MI lipid management and improving long-term outcomes. Among these, evolocumab has demonstrated consistent and robust efficacy in lowering LDL-C and reducing major adverse CV events in high-risk patients, making it a valuable option for those who do not achieve LDL-C goals with conventional therapy alone.

## Data availability statement

Not applicable.

## Ethics approval and consent to participate

Not applicable.

## Author contributions

Pravin K Goel, Aditya Kapoor, Kala Jeetender Jain, Rajesh Thachathodiyl, Vikas Reddy, Vivek Guleria, Neeraj Bhalla, and Shyam Sasidharan contributed to the conceptualization, data curation, formal analysis, funding acquisition, investigation, methodology, resources, supervision, validation, and visualization of the study. Aditya Kapoor prepared the original draft of the manuscript, and all authors contributed to the review and editing. All authors read and approved the final manuscript.

## Funding

This research received no external funding.

## Declaration of competing interest

The authors declare that they have no known competing financial interests or personal relationships that could have appeared to influence the work reported in this paper.

## Glossary

AACE: American Association of Clinical Endocrinology
ACC: American College of Cardiology
ACEP: American College of Emergency Physicians
ACS: Acute coronary syndrome
AHA: American Heart Association
apoER2: Apolipoprotein E receptor 2
ASCVD: Atherosclerotic cardiovascular disease
CD36: Cluster of differentiation 36
CI: Confidence interval
CK: Creatine kinase
CRP: C-reactive protein
CV: Cardiovascular
CVD: Cardiovascular disease
EARLY-STEMI: Early Evolocumab in Patients with ST-Segment Elevation Myocardial Infarction
EPICOR: Long-tErm follow-up of antithrombotic management Patterns In acute CORonary syndrome patients
EPIC-STEMI: Effects of Routine Early Treatment with PCSK9 Inhibitor in Patients Undergoing Primary Percutaneous Coronary Intervention for STEMI
ESC: European Society of Cardiology
EVACS: Evolocumab in Acute Coronary Syndrome
EVOPACS: EVOlocumab for Early Reduction of LDL-Cholesterol Levels in Patients With Acute Coronary Syndromes
FDA: United States Food and Drug Administration
FOURIER: Further Cardiovascular Outcomes Research with PCSK9 Inhibition in Subjects with Elevated Risk
GLAGOV: Global Assessment of Plaque Regression with a PCSK9 Antibody as Measured by Intravascular Ultrasound
HDL: High-density lipoprotein
HF: Heart failure
HIS: High-intensity statin
HR: Hazard ratio
HUYGENS: High-Resolution Assessment of Coronary Plaques in a Global Evolocumab Randomized Study
IL-17 A: Interleukin 17 A
LDL-C: Low-density lipoprotein cholesterol
LLT: Lipid-lowering therapy
LOX1: Lectin-like oxidised LDL receptor-1
Lp(a): Lipoprotein(a)
LRP1: Low-density lipoprotein receptor-related protein 1
MACE: Major adverse cardiovascular events
mAb: Monoclonal antibody
MI: Myocardial infarction
NSTEMI: Non-ST–segment elevation myocardial infarction
OLE: Open-label extension
OSLER: Open-Label Study of Long-Term Evaluation Against LDL-C
PACMAN-AMI: Effects of the PCSK9 Antibody Alirocumab on Coronary Atherosclerosis in Patients With Acute Myocardial Infarction
PAV: Percent atheroma volume
PCI: Percutaneous coronary intervention
PCSK9: Proprotein convertase subtilisin/kexin type 9
PCSK9i: Proprotein convertase subtilisin/kexin type 9 inhibitor
RECORD ACS-2: RECent trends in the pattern and lOng-term management stRategy of patients Diagnosed with Acute Coronary Syndrome
RR: Relative risk
SCAI: Society for Cardiovascular Angiography and Interventions
siRNA: Small Interfering RNA
STEMI: ST-segment elevation myocardial infarction
SWEDEHEART: Swedish Web-System for Enhancement and Development of Evidence-Based Care in Heart Disease Evaluated According to Recommended Therapies
VALIANT: Valsartan in Acute Myocardial Infarction Trial
VCU-AlirocRT: Virginia Commonwealth University Alirocumab Randomised Trial
VESALIUS-REAL: Real-world Effectiveness of Evolocumab in High-Risk Patients With Atherosclerosis
